# Flavones and Aminoflavones Increase the Cytotoxicity of NK Cells in Human Non‐Small Cell Lung Cancer

**DOI:** 10.1111/jcmm.71055

**Published:** 2026-02-13

**Authors:** Ping‐Chih Hsu, Chih‐Cheng Chien, Yang‐Je Cheng, Chuan‐Hsin Chang, Tzenge‐Lien Shih

**Affiliations:** ^1^ Division of Thoracic Medicine, Department of Internal Medicine Chang Gung Memorial Hospital at Linkou Taoyuan Taiwan; ^2^ Department of Medicine, College of Medicine Chang Gung University Taoyuan Taiwan; ^3^ Institute of Ecology and Evolutionary Biology, National Taiwan University Taipei City Taiwan; ^4^ Department of Chemistry Tamkang University New Taipei City Taiwan; ^5^ Department of Research Taipei Tzu Chi Hospital, Buddhist Tzu Chi Medical Foundation New Taipei City Taiwan; ^6^ Research Center for Chinese Herbal Medicine, Graduate Institute of Healthy Industry Technology, College of Human Ecology, Chang Gung University of Science and Technology Taoyuan Taiwan

**Keywords:** aminoflavone, anti‐cancer, flavone, lung cancer, natural killer

## Abstract

Natural flavonoids (flavones) and synthetic aminoflavones are known for their anti‐cancer properties; however, their immunomodulation ability has been largely unexplored. This study determined that synthetic flavones and aminoflavones modulate the cytotoxicity of natural killer (NK) cells against lung cancer cells. Notably, flavones 2, 3, and 6 and aminoflavone 8 were shown to increase the cytotoxicity of NK‐92MI cells against A549 lung cancer cells without adversely affecting MRC5 normal cells. Aminoflavone 8 enhanced NK‐92MI cell cytotoxicity, as evidenced by the elevated expression of cytotoxic effectors, such as IFN‐γ, perforin, and granzyme B. Aminoflavone 8 also inhibited STAT3 phosphorylation in A549 lung cancer and NK‐92MI cells under co‐culture conditions. Moreover, aminoflavone 8 exhibited anti‐tumour effects in a lung cancer xenograft mouse model. Combined therapy with aminoflavone 8 and NK‐92MI cells had synergistic anti‐tumour effects without liver or kidney toxicity. Our analysis revealed that the amino group in the C6 position of aminoflavone 8 was crucial to the enhanced cytotoxicity of NK cells. These findings suggest that aminoflavone 8 can potentiate NK cell cytotoxicity against lung cancer cells, highlighting its potential as a novel therapeutic agent for the treatment of lung cancer.

**Abbreviations**
ATCC: American type culture collection
BUN: blood urea nitrogen
CRE: creatinine
GOP: glutamic‐oxaloacetic transaminase
GPT: glutamic‐pyruvic transaminase
IFN‐γ: interferon‐γ
KIRs: killer inhibitory receptors
LDH: lactate dehydrogenase
MICA: MHC class I chain‐related proteins A
NK: natural killer
NSCLC: non‐small cell lung cancer
STAT3: signal transducer and activator of transcription 3
ULBPs: UL16‐binding proteins
WST‐1: 2‐(4‐Iodophenyl)‐3‐(4‐nitrophenyl)‐5‐(2,4‐disulfophenyl)‐2H‐tetrazolium monosodium salt

## 1 Introduction

**FIGURE 1:**
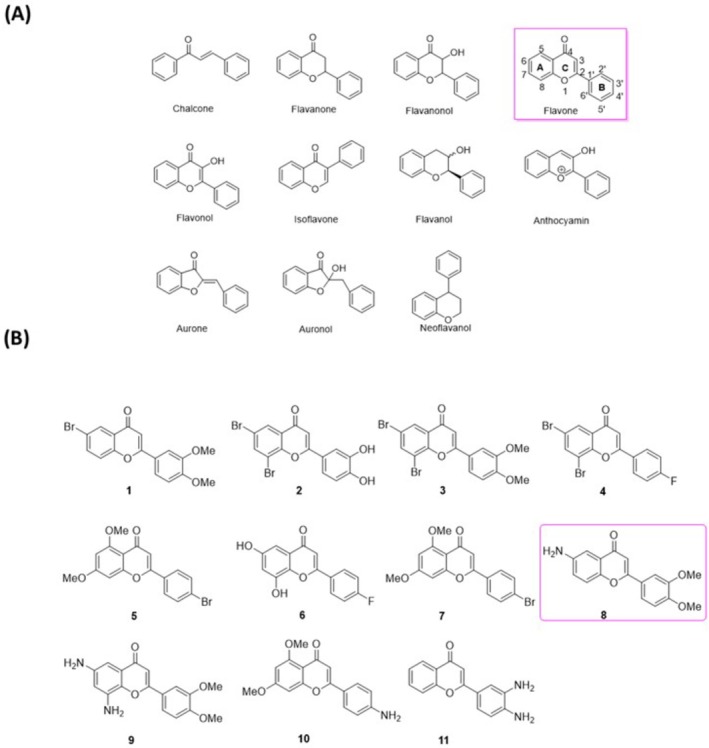
(A) Flavonoids subfamily; (B) novel synthetic flavones and aminoflavones.

The term flavones refers to a highly abundant subfamily of flavonoids with 11 members (Figure 1A) [1]. Flavonoids generally function as secondary metabolites in higher plants, including fruits, tea, wine, and vegetables [2]. These compounds exhibit various biological properties, including antioxidant, anti‐inflammatory, anti‐microbial, and anti‐cancer activities [2, 3]. It has been established that the consumption of flavonoids from dietary sources can be beneficial to human health by preventing various diseases.

As shown in Figure 1A, the structure of flavones consists of two phenyl groups (denoted A and B) connected to a heterocyclic ring at position 2 (denoted C). Naturally occurring flavones typically contain hydroxy groups, particularly at positions 5 and 7 of the A ring and at positions 3′,4′ or 3′,4′,6′ of the B ring [1]. Aminoflavones are relatively rare synthetic flavones with amino substitutions. Two aminoflavone prodrugs known as NSC 686288 and NSC 710464 (Figure S1) have shown promising inhibitory effects on MCF7 human breast cancer cells and other cell lines [4, 5, 6]. Moreover, researchers have synthesised a series of N‐benzyl derivatives of 6‐aminoflavone with anti‐cancer abilities against both human breast and lung cancer cell lines through the inhibition of topoisomerase II activity [7]. Aminoflavones have also been studied in the context of neurodegenerative diseases [8, 9]. Notably, in a breast cancer mouse model, the aminoflavone prodrug NSC 710464 was shown to act as an immunomodulator by reducing the quantity of myeloid‐derived suppressor cells and increasing the anti‐tumour M1‐macrophage profile, resulting in anti‐cancer effects [10].

Lung cancer is a leading cause of cancer‐related death, ranked second in terms of cancer diagnosis and third in terms of incidence [11]. According to the Global Surveillance of Trends in Cancer Survival 2000–14 report, the 5‐year survival rate for patients diagnosed with lung cancer is only 10% to 20% in most countries [12]. Depending on the stage of lung cancer, patients may qualify for specific multimodal treatments, including surgery, radiation, chemotherapy, immunotherapy, targeted therapy, and vaccines [11, 12]. Unfortunately, lung cancer patients inevitably develop drug resistance [13, 14, 15]. Novel compounds have been developed to modulate the tumour microenvironment and suppress tumour growth/progression without the toxicity profiles of previous drugs. These compounds are emerging candidates for development as standalone cancer drugs or adjuvant therapies.

Human natural killer (NK) cells play a crucial role in defence mechanisms in the innate immune system against viral infections and malignant cells [16, 17, 18]. The anti‐tumour activity of NK cells is mediated through various mechanisms, including direct antibody‐dependent cellular cytotoxicity, the release of cytotoxic molecules, and the secretion of immunostimulatory cytokines [19, 20, 21] and chemokines [22]. However, cancer cells have developed strategies to evade NK cell attacks, leading to NK cell dysfunction in many cancer patients. This dysfunction is characterised by several factors, including limited proliferation and low NK cell concentrations in peripheral blood and tumour tissues, reduced cytotoxicity, impaired expression of activating receptors and natural cytotoxicity receptors, overexpression of inhibitory receptors, presence of immune checkpoint inhibitors, and impaired cytokine production [23, 24, 25]. Furthermore, the ligands for the NK cell activating receptor in tumour cells have been associated with a favourable prognosis for patients with non‐small cell lung cancer (NSCLC), which means that they also have predictive value for the clinical outcomes of cancer patients [26, 27, 28].

Our research group previously reported on the synthesis of flavones 1–7 and aminoflavones 8–11 [29] (Figure 1B); however, those compounds had not yet undergone biological assays. Our aim in the current study was to explore the biological activities of these flavones and aminoflavones as candidates for drug development. We investigated whether these compounds could inhibit the proliferation of lung cancer cells or modulate the anti‐tumour immune response by enhancing the anti‐tumour activity of NK cells against human lung cancer cells. We first identified hit compounds with selective anti‐NSCLC activities and/or the ability to stimulate the anti‐cancer activity of NK cells against human lung cancer cells. Under co‐culture conditions, some of the compounds were shown to regulate the expression of cytotoxic factors, thereby activating receptors and their ligands as well as immune checkpoint inhibitors in NK cells or human lung cancer cells. Our results indicate the potential of these flavones and aminoflavones as immunostimulatory agents for NK cells in tumour treatment.

## 2 Materials and Methods

### 2.1 Chemicals and Reagents

The synthesis and spectroscopic data of compounds 1–11 have previously been reported [29]. 2‐(4‐Iodophenyl)‐3‐(4‐nitrophenyl)‐5‐(2,4‐disulfophenyl)‐2H‐tetrazolium monosodium salt (WST‐1) was obtained from Dojindo (Kumamoto, Japan). Fluorescein isothiocyanate (FITC)‐labelled anti‐CD56 antibody (5 μL/million cells, # 318303) and Brilliant Violet 421‐labelled anti‐perforin antibody (5 μL/million cells, # 308121) or PE‐labelled‐granzyme B antibody (5 μL/million cells, #372203) used in flow cytometry analysis were purchased from BioLegend (San Diego, CA, USA). Anti‐STAT3 antibody (1:1000, #12640), anti‐phospho‐Stat3 (Tyr705) antibody (1:1000, #9131), and anti‐glyceraldehyde‐3‐phosphate dehydrogenase (GADPH) antibody (1:1000, #2118) were purchased from Cell Signalling Technology (Danvers, MA, USA). Horseradish peroxidase (HRP) anti‐rabbit IgG (1:5000, #32460) and HRP anti‐mouse IgG (1:200,000, #31430) were purchased from Thermo Fisher Scientific (Waltham, MA, USA). Other chemicals and reagents were obtained from Sigma (St. Louis, MO, USA).

### 2.2 Cell Culture

Cells from the human lung cancer cell line A549 were obtained from the American Type Culture Collection (ATCC), and H1975 cells were kindly provided by Dr. Pan‐Chyr Yang of National Taiwan University in Taiwan; normal cell line: MRC5 cells were kindly provided by Dr. Chi‐Yuan Chen of Chang Gung University of Science and Technology in Taiwan. Those cells were maintained in RPMI‐1640 medium or Eagle's Minimum Essential Medium (MEM) (GIBCO, CA, USA) supplemented with 10% fetal bovine serum (FBS; GIBCO, CA, USA) and 1× antibiotic‐antimycotic solution (GIBCO, CA, USA). Human NK cell line: NK‐92MI cells were maintained in α‐MEM without ribonucleosides or deoxyribonucleosides (GIBCO, CA, USA) with 2 mM L‐glutamine and 1.5 g/L sodium bicarbonate. The medium was also supplemented with 0.2 mM inositol, 0.1 mM 2‐ME, 0.02 mM folic acid, 12.5% FBS, and 12.5% horse serum (GIBCO, CA, USA). All cells are cultured at 37°C in an incubator containing 5% CO_2_.

### 2.3 Cellular Viability Assay

Cell viability was assessed using a WST‐1 assay. Briefly, 2 × 10^3^–2 × 10^4^ cells (lung cancer, normal, or NK cells) were plated in 96‐well culture plates at a final volume of 100 μL/well. After 24 h, cells were treated with various concentrations of flavones or aminoflavones. The treated medium was then centrifuged at 200 × *g* for 5 min, and the supernatant was incubated with WST‐1 reagent for 2 h at 37°C, whereupon the absorbance was measured at 450 nm and the background absorbance was measured at 620 nm.

### 2.4 Selectivity Index (SI)

The SI was calculated to assess the compound's preferential cytotoxicity toward cancer cells over normal cells. SI was defined as the ratio of the IC_50_ value in normal human lung fibroblasts (MRC‐5) to the IC_50_ value in lung cancer cell lines (A549 and H1975). An SI value greater than 3.0 indicates high selectivity for cancer cells over normal cells, suggesting potential therapeutic relevance [30, 31, 32].

$$\text{Selectivity Index}\;\left(\mathrm{SI}\right)=\frac{\text{Cytotoxicity toward normal cells}}{\text{Cytotoxicity toward lung cancer cells}}$$

### 2.5 Cytotoxicity Assay

The cytotoxic activity of NK‐92MI cells was assessed using CytoTox 96 Non‐Radioactive Cytotoxicity Assay (Promega, Madison, WI, USA) in accordance with the manufacturer's protocol. Briefly, NK‐92MI cells were co‐cultured with target cells (lung cancer or normal cells), with or without compound pre‐treatment (10 μM for 24 h), at various effector‐to‐target (E:T) ratios: 10:1, 5:1, and 1:1. The cells were seeded in round‐bottom 96‐well plates with a final volume of 100 μL per well, gently mixed, centrifuged at 250 × *g* for 5 min, and incubated at 37°C with 5% CO_2_ for either 4 h or 24 h. After incubation, 50 μL of the supernatant from each well was transferred to the corresponding well of a flat‐bottom 96‐well enzymatic assay plate. To each well, 50 μL of the CytoTox 96 Reagent was added, followed by incubation at room temperature for 30 min under protection from the light. After adding 50 μL of Stop Solution to each well, the absorbance was measured at 490 nm. The cytotoxicity of each effector as a function of the target cell ratio was calculated as a percent using the following equation:

$$\frac{\left(\text{Experimental culture medium background}\right)-\left(\text{Effector cell spontaneous release culture medium background}\right)-\left(\text{Target spontaneous release culture medium background}\right)}{\left(\text{Target maximum release volume correction control}-\text{Target spontaneous release culture medium background}\right)}\times 100.$$

### 2.6 IFN‐γ Elisa

The secretion of IFN‐γ from NK cells was estimated using an NK VUE Kit (NKMAX, Seongnam‐si, Korea) in accordance with the manufacturer's instructions. After centrifuging at 11,500 × *g* for 1 min at room temperature, the supernatant was transferred into diluent‐loaded ELISA wells and incubated at room temperature for 1 h. Following the removal of unbound material through washing, IFN‐γ was assessed using anti‐IFN‐γ antibodies conjugated to HRP for 1.5 h. A further wash step was performed to eliminate unbound antibody‐HRP complex, and 3,3′, 5,5′‐tetramethylbenzidine (TMZ) substrate was then added to each well and left to stand for 30 min, after which the absorbance was measured at 450 nm.

### 2.7 Flow Cytometry

Intracellular perforin and granzyme B production were detected by fixing cells for permeabilization using a BD Cytofix/Cytoperm Fixation/Permeabilization Solution Kit (BD Biosciences, San Jose, CA, USA) in accordance with the manufacturer's instructions. Briefly, the cells were stained using FITC‐labelled anti‐CD56 and PE‐labelled perforin or granzyme B antibodies (BioLegend, San Diego, CA, USA). Flow cytometry analysis was performed using a FACSCalibur flow cytometer (Becton Dickinson, Franklin Lakes, NJ, USA). The fluorescence intensity of at least 1 × 10^5^ cells was recorded and analysed using CellQuest software (Becton Dickinson, Franklin Lakes, NJ, USA).

### 2.8 RNA Preparation and Quantitative Reverse Transcription‐PCR

RNA was extracted using Trizol (Invitrogen, Carlsbad, CA, USA) and chloroform and then precipitated using isopropanol in accordance with the manufacturer's recommendations. The RNA concentration was quantified using a Nano100 Micro‐Spectrophotometer (CLUBIO; Taipei, Taiwan). cDNA synthesis was then performed using SuperScript IV Reverse Transcriptase (Invitrogen, Carlsbad, CA, USA) and amplified using a spectrofluorometric thermal cycler (iCycler; Bio‐Rad Laboratories). mRNA expression by A549 and NK‐92MI cells was assessed via qRT‐PCR in accordance with a well‐established protocol using *GAPDH* as the internal control [33]. The fluorescence emission by iQ SYBR Green Supermix (Bio‐Rad, Hercules, CA, USA) was measured using a CFX Connect Real‐Time PCR detection system. Relative gene expression was determined using the delta–delta‐Ct (ddCt) method, where Ct refers to the mean threshold cycle. All primers were purchased from MDBio Inc. (Taipei, Taiwan). The primers used for real‐time PCR experiments are listed in Table S1.

### 2.9 Western Blotting Analysis

Lung cancer cells pre‐treated with aminoflavone 8 were then co‐cultured with NK‐92MI cells. The cells were lysed with sample buffer and heat‐treated at 95°C–100°C for 5 min, followed by centrifugation to remove cell debris. The supernatant was then used to assess STAT3 expression or phosphorylation via immunoblotting analysis using corresponding secondary rabbit antibodies. Immunoreactive bands were visualised using HRP and analysed using UVP Biospectrum (UVP, Upland, CA, USA).

### 2.10 Animal Study

This study established a lung cancer xenograft mice model involving non‐obese diabetes/severe combined immune deficiency (NOD/SCID) mice (BioLasco, Taiwan) aged 5–6 weeks and weighing 20–25 g. The animals were maintained in plastic cages with free access to water and food. The experiment began with the subcutaneous injection of 2 × 10^6^ A549 cells in the right‐side dorsa. When the tumours reached a volume range of 50 mm^3^, the mice were segregated into four groups (control, aminoflavone 8, NK‐92MI, and NK‐92MI + aminoflavone 8). Aminoflavone 8 (5 mg/kg) was injected intraperitoneally five times per week over a period of 40 days. NK‐92MI cells (5 × 10^5^ cells/animal) were injected intravenously once per week for 4 weeks. Tumour size was measured weekly. The mice were sacrificed when the tumours reached the maximum allowed volume of 1500 mm^3^. Serum blood urea nitrogen (BUN) levels as well as creatinine (CRE), glutamic‐oxaloacetic transaminase (GOT), and glutamic‐pyruvic transaminase (GPT) levels were obtained using an automated clinical chemistry analyser (Dri‐Chem NX500i, Fujifilm, Japan). The animal experiment in this study was performed in accordance with US guidelines (NIH publication #85–23, revised in 1985)^33^ and had been approved by the Institutional Animal Care and Use Committee of Chang Gung Memorial Hospital, Taiwan (IACUC approval no. 202309201).

### 2.11 Statistical Analysis

All experiments were performed at least three times, and the data were expressed as means ± standard error (SE). Statistical analysis was conducted by either Student's *t*‐test or ANOVA (Prism, GraphPad Software 9.0.2, San Diego, CA, USA). *p* < 0.05 was considered statistically significant.

## 3 Results

### 3.1 Cytotoxic Effects of Candidate Compounds on Lung Cancer Cells

The anti‐cancer effect of the candidate compounds was assessed in vitro by examining the viability of lung cancer cell lines, A549 (with EGFR wild‐type mutations) and H1975 (with EGFR T790M and L858R mutations), at the 72 h‐time point. As shown in Table 1a, aminoflavone 8 demonstrated moderate anti‐cancer effects against A549 lung cancer cells, as indicated by a SI of > 3.00 and an IC_50_ value of 23.14 ± 1.29 μM. Compounds 6 and 11 also exhibited moderate antiproliferative effects against A549 cells, as indicated by SI values of > 3.00 and IC_50_ values of 24.20 ± 1.50 μM and 34.38 ± 1.57 μM, respectively. Aminoflavone 8 exhibited moderate anti‐tumour effects against H1975 lung cancer cells, with a relatively lower SI value of 1.81 (Tables 1a and 1b).

**TABLE 1a:** Antiproliferative and cytotoxic effects of candidate compounds against lung cancer cells.

Sample No.	A549 (EGFR^wt^)			SI	H1975 (EGFR T790M/L858R)			SI
	IC_50_ (μM)^a^				IC_50_ (μM)^a^			
1		> 100^c^		–^d^		> 100^c^		–^d^
2	151.50	±	0.20	0.25	214.00	±	0.56	0.17
3		> 100^c^		–^d^	> 100^c^			–^d^
4		> 100^c^		–^d^	> 100^c^			–^d^
5		> 100^c^		–^d^	> 100^c^			–^d^
6	24.20	±	1.50	4.53	77.27	±	0.97	1.42
7		> 100^c^		–^d^		> 100^c^	.	–^d^
8	23.14	±	1.29	3.03	38.65	±	1.48	1.81
9	56.13	±	1.35	> 1.78	72.78	±	1.39	> 1.37
10	130.70	±	0.78	> 0.77	82.92	±	0.55	> 1.21
11	34.38	±	1.57	> 2.91	53.84	±	1.16	> 1.86
Doxorubicin^b^	1.66	±	0.51	0.91	0.78	±	0.77	1.94

*Note:* Results are presented as means ± standard error (SE) (*n* = 3).

^a^ Concentration necessary for 50% inhibition (IC_50_).

^b^ Doxorubicin was used as a positive control.

^c^ Values reported as > 100 μM indicate that 50% inhibition was not reached at the highest tested concentration.

^d^ “–” indicates not applicable due to lack of measurable IC_50_ in normal cells or cancer cells.

**TABLE 1b:** Antiproliferative effects of candidate compounds in MRC5 normal cells.

Sample No.	MRC5		
	IC_50_ (μM)^a^		
1	98.56	±	1.53
2		> 100^c^	
3	86.74	±	1.04
4	69.35	±	1.71
5	84.99	±	1.45
6	109.7	±	1.26
7	89.8	±	1.51
8	70.12	±	1.16
9		> 100^c^	
10		> 100^c^	
11		> 100^c^	
Doxorubicin^b^	1.51	±	0.78

*Note:* Results are presented as means ± standard error (SE) (*n* = 3).

^a^ Concentration necessary for 50% inhibition (IC_50_).

^b^ Doxorubicin was used as a positive control.

^c^ Values reported as > 100 μM indicate that 50% inhibition was not reached at the highest tested concentration.

### 3.2 Effects of Compounds on the Viability of NK Cells

**FIGURE 2:**
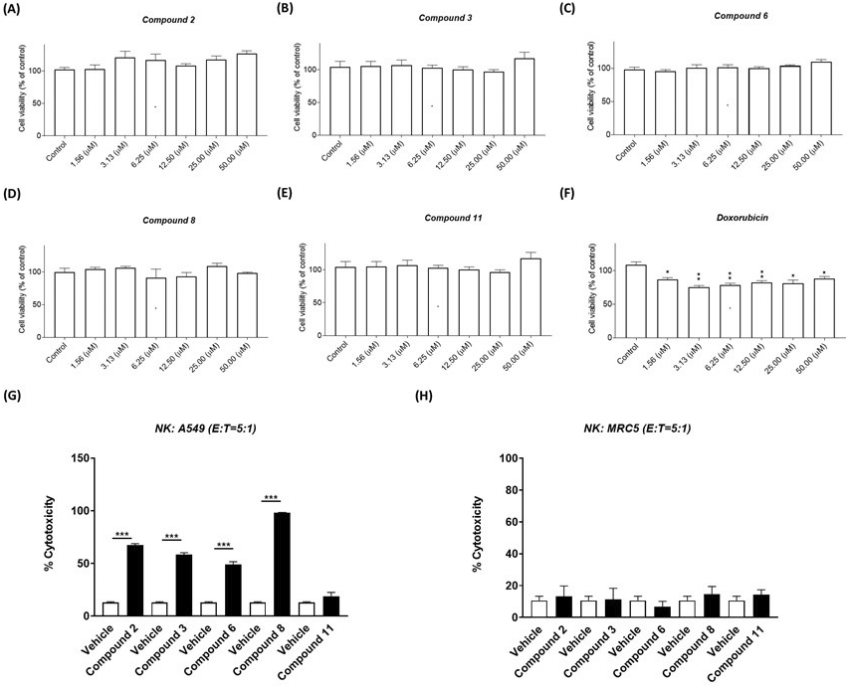
Novel flavones and aminoflavones enhanced the cytotoxicity of NK cells against lung cancer cells. The effect of the compounds on NK‐92MI cell viability was assessed by treating NK‐92MI cells with candidate compounds (A) 2, (B) 3, (C) 6, (D) 8, and (E) 11 or (F) doxorubicin for 24 h. NK‐92MI cell viability was measured using a WST‐1 assay. LDH cytotoxicity assays were also used to measure the cytolysis of NK‐92MI cells against A549 lung cancer cells (G) or MRC5 normal cells (H) treated with the novel synthetic compounds or vehicle (as a control group). A549 lung cancer cells or MRC5 normal cells were treated with the compounds or a vehicle for 24 h and then co‐cultured with NK‐92MI cells at an E:T ratio of 5:1 for an additional 4 h. The presented data are the means ± SE of three independent experiments. Statistical analysis revealed significant differences (***p < 0.001) between the experimental groups and control group.

As shown in Figure 2A–F, this study assessed the impact of compounds 2, 3, 6, 8, and 11 and the commonly used chemotherapy drug doxorubicin (at various concentrations) on the viability of NK‐92MI cells. Compounds 2, 3, 6, 8, and 11 had no significant effect on cell viability at concentrations below 50 μM. Doxorubicin had a notable effect on cell viability even at a low dose of 1.5 μM. Consequently, all subsequent experiments involving these compounds were conducted using the maximum non‐toxic concentration, which was determined to be 10 μM.

### 3.3 Effects of Candidate Compounds on NK Cytotoxicity Against Lung Cancer Cells

The impact of the candidate flavones and aminoflavones on the cytotoxicity of NK cells against lung cancer cells was assessed by performing lactate dehydrogenase (LDH) release assays using NK cells co‐cultured with A549 lung cancer cells that had been pre‐treated with candidate compounds (2, 3, 6, 8, or 11) at a concentration of 10 μM. The effector‐to‐target (E/T) ratio was set at 5:1, and DMSO at a final concentration of 0.1% was used as a control. As shown in Figure 2G, treatment with flavones 2, 3, and 6 or aminoflavone 8 at a dose of 10 μM (E/T ratio 5:1) significantly enhanced NK cytotoxicity against the target cancer cells (A549 lung cancer cells). Importantly, these compounds had no observed effects on NK cytotoxicity against normal cells (MRC5 cells) (Figure 2H).

**FIGURE 3:**
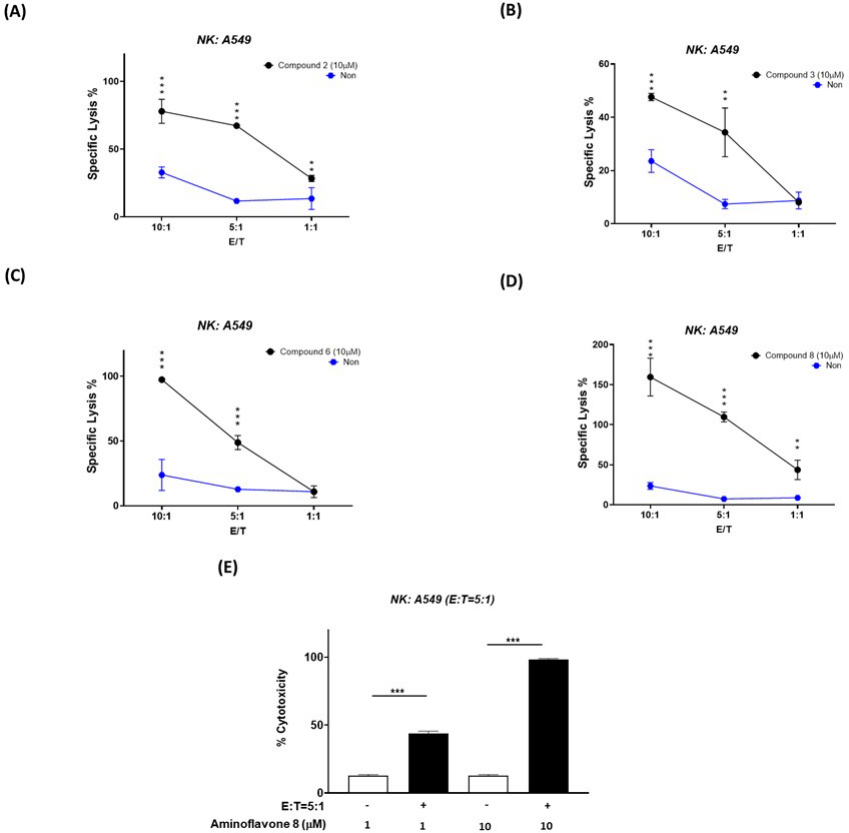
The effect of novel flavones and aminoflavones on NK cell–mediated cytotoxicity against human lung cancer and normal cells under various E:T ratios and compound concentrations. NK‐92MI cells were co‐cultured with A549 lung cancer cells and treated with compounds 2 (A), 3 (B), 6 (C), or 8 (D) at 10 μM, using effector‐to‐target (E:T) ratios of 1:1, 5:1, or 10:1. NK‐92MI‐mediated cytotoxicity at each E:T ratio without compound treatment was included as a control (i.e., baseline NK cell cytotoxicity against A549). (E) A549 cells were pretreated with aminoflavone 8 at either 1 μM or 10 μM and then co‐cultured with NK‐92MI cells at an E:T ratio of 5:1. The untreated NK‐92MI/A549 co‐culture as the control group at a 5:1 ratio shown in previous panels. Cytotoxicity was measured using the LDH assay. The presented data are the means ± SE of three independent experiments. Statistical analysis revealed significant differences (**p < 0.01; ***p < 0.001) between the experiment groups and control group.

This study also examined the effects of flavones 2, 3, and 6 and aminoflavone 8 in modulating NK cell cytotoxicity at various E/T ratios (10:1, 5:1, or 1:1). As shown in Figure 3A–D, compounds 2, 3, 6, and 8 increased NK cell cytotoxicity against A549 lung cancer cells at various E/T ratios. Among the candidate compounds, aminoflavone 8 demonstrated the greatest potency in inducing NK cell cytotoxicity against A549 lung cancer cells. Aminoflavone 8 was also shown to enhance the cytotoxic ability of NK‐92MI cells against A549 lung cancer cells in a dose‐dependent manner (Figure 3E).

### 3.4 Effect of Aminoflavone 8 on the Expression of Cytotoxic Effectors in NK Cells Under Co‐Culture Conditions

**FIGURE 4:**
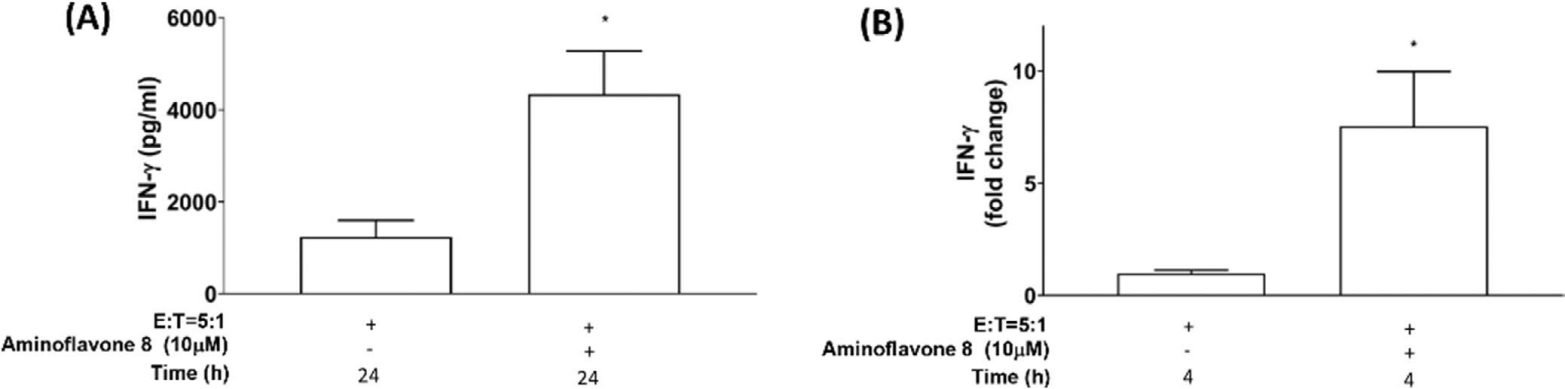
Aminoflavone 8 increased IFN‐γ production in human NK cells. NK cells were co‐cultured with A549 in the presence or absence of aminoflavone 8 (10 μM) for 24 h to determine IFN‐γ secretion levels using ELISA (A). mRNA levels of *IFN‐γ* in NK‐92MI cells (E:T = 5:1) were determined by qRT‐PCR analysis (normalised to the quantity of *GAPDH* mRNA) (B). Bars indicate the mean ± SE of three independent experiments with significant differences indicated at *p < 0.05 versus the corresponding control.

IFN‐γ is a cytotoxic cytokine produced by NK cells that can inhibit tumour cell growth, induce cancer cell death, and modulate the tumour microenvironment [19, 20, 21]. The results of protein and gene expression analysis revealed that under co‐culture conditions, aminoflavone 8 significantly induced IFN‐γ secretion and *IFN‐γ* gene expression levels in NK‐92MI cells (Figure 4).

**FIGURE 5:**
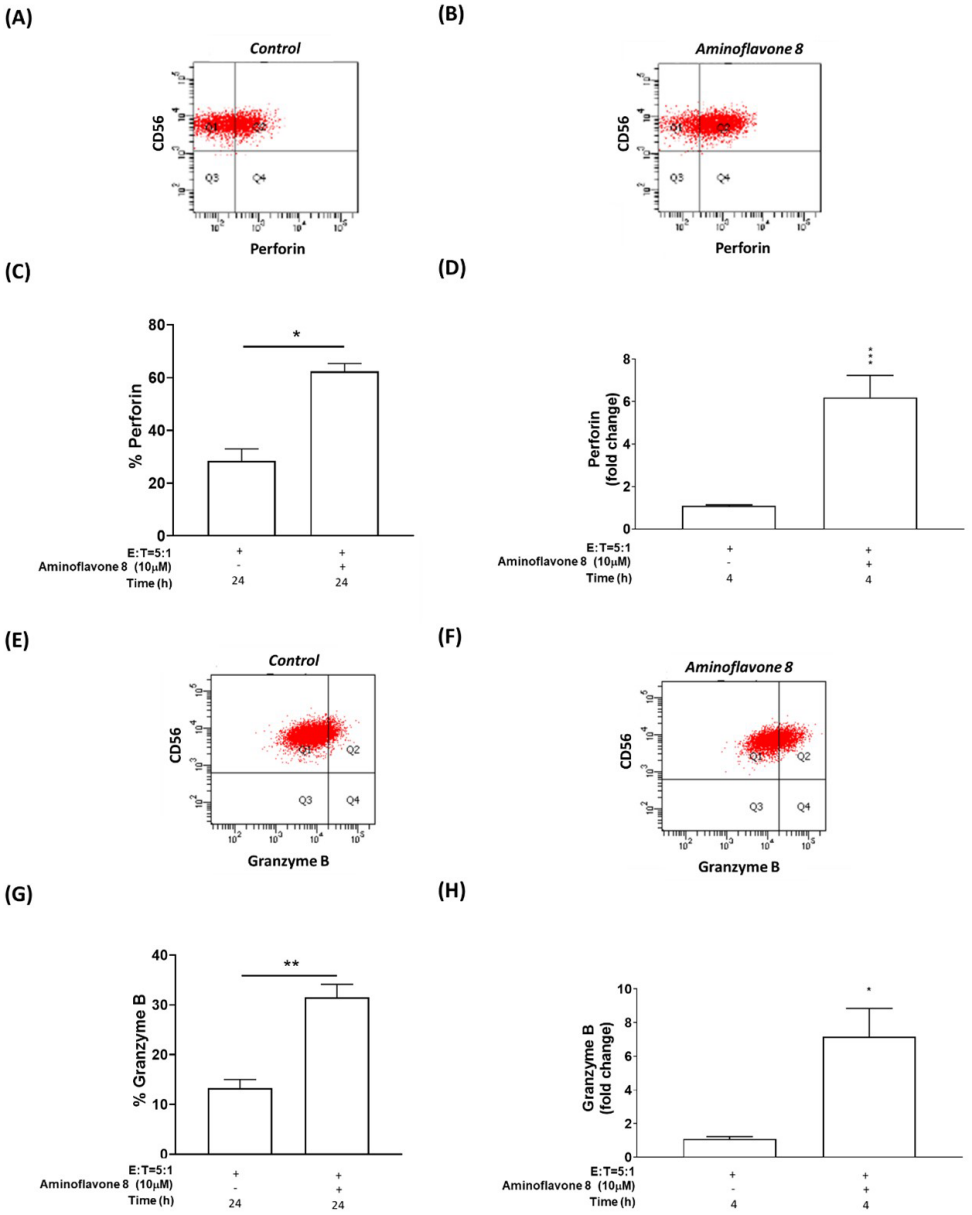
Perforin and Granzyme B expression in NK cells treated with aminoflavone 8 and co‐cultured with A549 lung cancer cell lines. Protein expression levels of perforin and granzyme B were evaluated in the absence of aminoflavone 8 (A, E) and in the presence of aminoflavone 8 (B, F). Perforin and granzyme B productions in CD56^+^ NK‐92MI were shown as a percentage in panels (C) and (G), respectively. mRNA levels of *perforin* (D) and *granzyme B* (H) in NK‐92MI cells (E:T = 5:1) were determined by qRT‐PCR (normalised to the amount of *GAPDH* mRNA). The bars in the figure represent mean ± SE. *p < 0.05; **p < 0.01; ***p < 0.001, versus the model group.

By functioning as a cytotoxic cytokine, IFN‐γ also works with granzyme B and perforin to promote apoptosis in tumour cells [34, 35, 36]. Perforin plays a pivotal role in NK cell‐mediated regulation of tumour growth and metastasis [36, 37, 38]. The release of cytolytic effectors (such as perforin and granzyme B) from NK cells (referred to as degranulation) increases cytotoxicity against target cells [36, 37, 38]. Protein expression results in the current study revealed that aminoflavone 8 treatment induced the production of perforin and granzyme B in NK‐92MI cells co‐cultured with A549 lung cancer cells (Figure 5A–C,E–G, respectively). Consistent with our previous results, mRNA expression levels revealed that aminoflavone 8 induced a significant increase in the gene expression of *perforin* and *granzyme B* in NK‐92MI cells under co‐culture conditions (Figure 5D,H, respectively).

### 3.5 Aminoflavone 8 Decreased the Activation of STAT3

**FIGURE 6:**
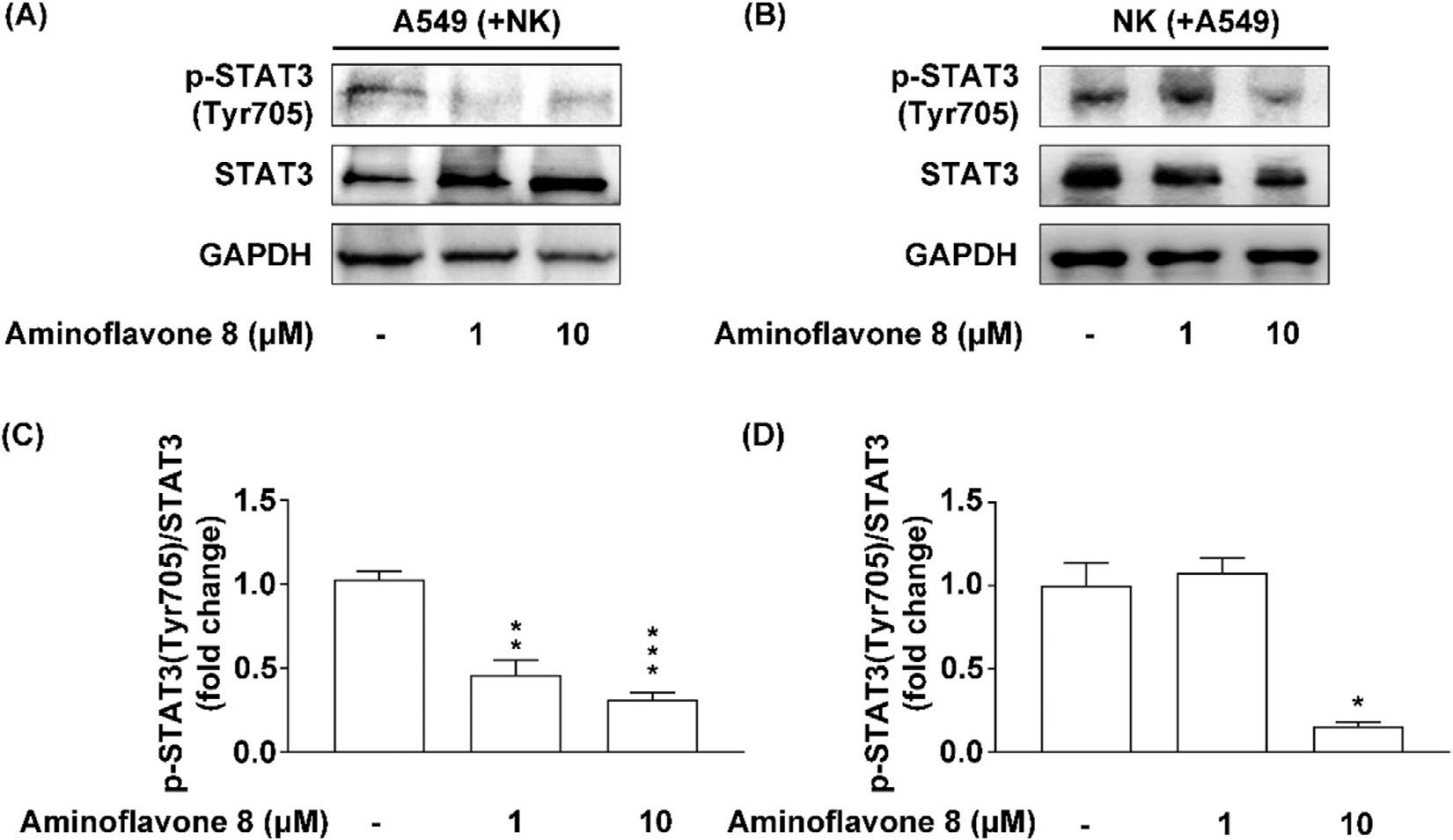
Treatment with aminoflavone 8 inhibited the phosphorylation of STAT3 at Tyr705 in A549 lung cancer cells and NK‐92MI cells under co‐culture conditions. (A–C) A549 cells or (D–F) NK‐92MI cells were treated with either DMSO (0.1%, as a control) or various concentrations of aminoflavone 8 (1 or 10 μM) for 24 h and then co‐cultured for another 4 h. The expression of STAT3 and its phosphorylation status were analysed via immunoblotting analysis using antibodies against total and phosphorylated STAT3. The data are presented as mean ± SE. (*n* = 3). Statistical analysis revealed significant differences (*p < 0.05; **p < 0.01; ***p < 0.001) compared to DMSO.

Several studies have demonstrated that STAT3 plays an important role in NK cell‐mediated tumour surveillance [39, 40, 41]. In the current study, we extended these investigations to determine whether aminoflavone 8 could regulate the STAT3 signalling pathways in NK‐92MI cells and A549 lung cancer cells under co‐culture conditions. Indeed, aminoflavone 8 significantly inhibited the phosphorylation of STAT3 at Tyr705 in both A549 cells (at doses of 1 and 10 μM, Figure 6A,C) and NK‐92MI cells (at a dose of 10 μM, Figure 6B,D).

### 3.6 In Vivo Evaluation Indicating the Therapeutic Efficacy of Aminoflavone 8 in Conjunction With NK Cells

**FIGURE 7:**
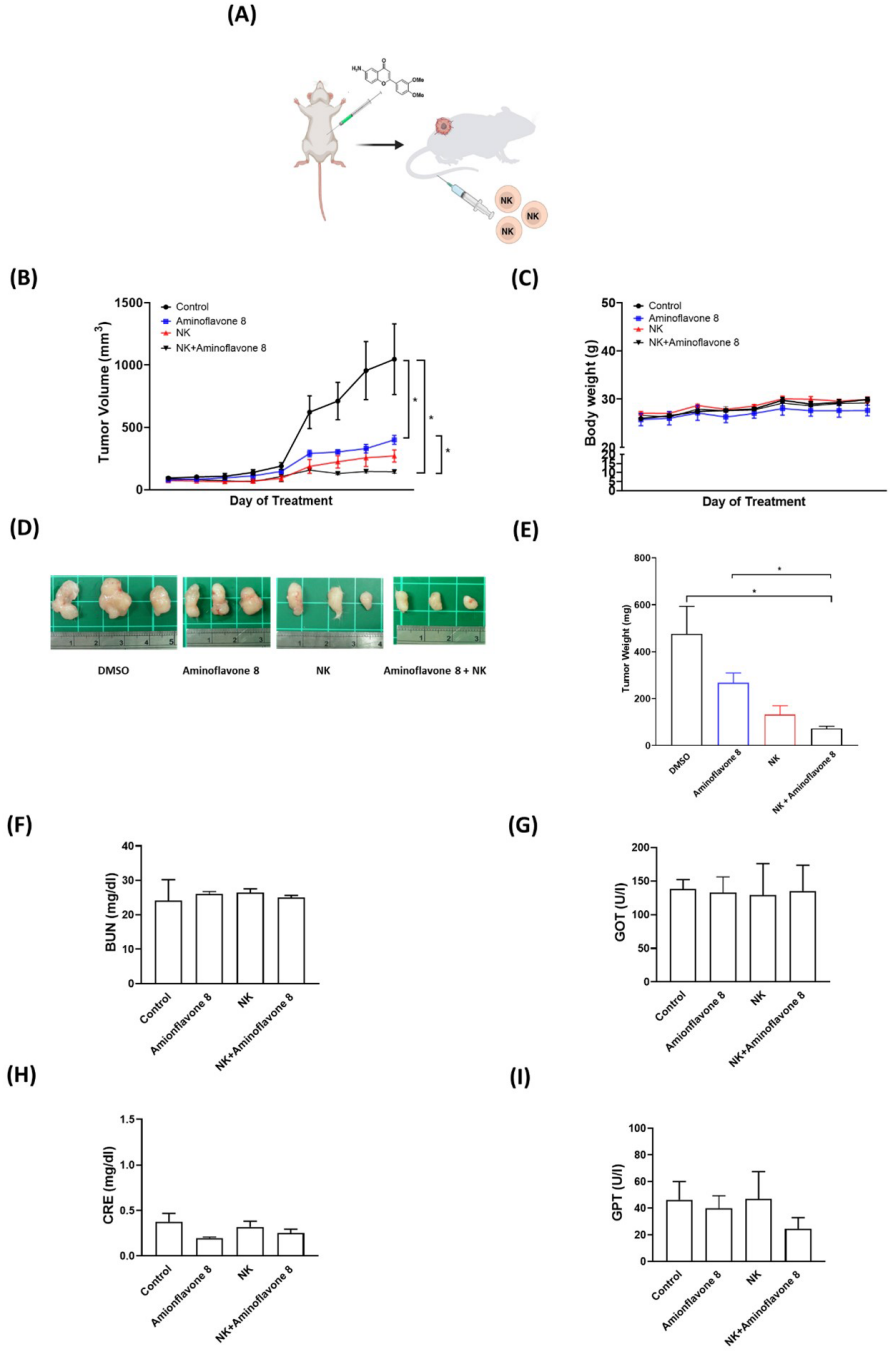
Administering aminoflavone 8 enhanced in vivo NK cell‐mediated anti‐tumour activity with no toxic effects on the liver or kidney. (A) A549‐injected NOD/SCID mice were treated with aminoflavone 8 (5 mg/kg), NK‐92MI (5 × 10^5^ cells), or NK‐92MI (5 × 10^5^ cells) + aminoflavone 8 (5 mg/kg) and assessed in terms of (B) tumour growth, (C) body weight, (D) tumour size, and (E) tumour weight. Tumour growth curves revealed that aminoflavone 8 and NK‐92MI + aminoflavone 8 treatments significantly inhibited tumour growth, compared to the control group. Individual or combined treatment with NK‐92MI and aminoflavone 8 did not affect body weight. Treatment effects on serum levels of BUN (F), CRE (G), GOT (H), and GPT (I) were measured using an automated clinical chemical analyser. Data are expressed as mean ± SE with significance differences indicated at *p < 0.05 versus the corresponding control.

We also assessed the in vivo anti‐tumour effects of NK‐92MI or aminoflavone 8 on lung cancer cells. Once the tumours reached a measurable size, each group received the vehicle control or compound 8 (5 mg/kg) five times per week for 40 days as well as NK‐92MI cells once per week for 4 weeks (Figure 7A). Treatment with aminoflavone 8 alone suppressed tumour growth, compared to the vehicle control (Figure 7B,D). Aminoflavone 8 also enhanced the anti‐tumour activity of NK‐92MI cells in vivo (Figure 7B,D,E). Treatment with NK‐92MI, aminoflavone 8, or a combination of the two did not have a significant effect on body weight (Figure 7C) or serum levels of BUN (Figure 7F), CRE (Figure 7G), GOT (Figure 7H), or GPT (Figure 7I), indicating that these treatments were non‐toxic to the liver and kidney.

## 4 Discussion

This study assessed various synthetic flavones and aminoflavones in terms of anti‐lung cancer activities against A549 and H1975 lung cancer cells with a focus on their ability to enhance the anti‐tumour activity of NK cells against A549 cells. The flavones and aminoflavones showed only moderate anti‐cancer effects when tested directly on lung cancer cells; however, this study revealed for the first time that aminoflavone 8 could enhance the cytotoxicity of NK‐92MI cells against A549 cells, without adverse effects on normal MRC5 cells.

The enhancement of anti‐cancer effects was associated with the increased expression of several important factors in NK cells at the gene or protein level, including cytotoxic effectors (such as IFN‐γ, perforin, and granzyme B) and activating receptors (such as *NKG2D*). Following treatment with aminoflavone 8, A549 lung cancer cells exhibited upregulated gene expression of ligands, including major histocompatibility complex class I‐related chain A (*MICA*) and UL16‐binding proteins (ULBPs) 1, 2, and 3 (*ULBP1*, *2*, and *3*) genes. Aminoflavone 8 also effectively inhibited STAT3 phosphorylation in A549 lung cancer cells and NK‐92MI cells under co‐culture conditions. In an animal model, aminoflavone 8 or NK‐92MI cells alone suppressed tumour growth in vivo. Moreover, combining aminoflavone 8 with NK‐92MI cells had a synergistic effect on anti‐tumour activity. The amino group at the C6 position of the A ring in aminoflavone 8 was shown to play a crucial role in enhancing NK cell cytotoxicity against lung cancer cells. This suggests that aminoflavone 8 could potentially be used as a novel therapeutic agent for the treatment of lung cancer.

NK‐92 cells are an established NK cell line derived from a male patient with non‐Hodgkin's lymphoma [42]. These cells appear to be a safe and potentially beneficial therapy for hematologic cancer, renal cell carcinoma, and advanced lung cancer [43, 44]. Note that NK‐92 cells have received approval for clinical application by the US Food and Drug Administration [45, 46]. These cells exhibit an atypical receptor expression profile, with the relatively high expression of several activating receptors (such as NKG2D, NKp30, and NKp46), while lacking most of the clonally expressed killer inhibitory receptors (KIRs) with the exception of KIR2DL4, which is typically found on normal NK cells [42, 47]. The NK‐92MI cells used in this study were derived from NK‐92 cells. The two cell types share similar biological functions; however, NK‐92MI cells do not require IL‐2 for growth [48]. Our results revealed that the novel synthetic flavones 2, 3, and 6 and aminoflavone 8 significantly enhanced the cytolytic activities of NK‐92MI cells against A549 lung cancer cells. Under co‐culture conditions, aminoflavone 8 affected cytotoxicity‐related markers in NK‐92MI cells and A549 cells.

In the tumour microenvironment, cancer cells can become resistant to NK cells, as a result of decreased cytotoxicity, defective expression of activating receptors (e.g., NKG2D), overexpression of immune checkpoint inhibitors (e.g., PD‐L1), or defective production of cytokines (e.g., IFN‐γ) or granular cytotoxic effectors (e.g., perforin and granzyme B) [23, 24, 25]. The expression of MHC class I chain‐related proteins A/B (MICA/B) is associated with a good prognosis in patients with NSCLC and predictive of clinical outcomes in cancer patients. These proteins function as ligands for the NK cell‐activating receptor NKG2D [26, 28]. Researchers have reported that high ULBP expression levels (ULBP1, ULBP2, and ULBP3) are associated with improved survival outcomes among patients with NSCLC [27], ovarian cancer [49], and breast cancer [50]. In the current study, treatment with aminoflavone 8 significantly induced the expression of IFN‐γ (Figure 4), perforin (Figure 5A–D), granzyme B (Figure 5E–H), and *NKG2D* (Figure S2) in NK‐92MI cells. Aminoflavone 8 treatment also led to an increase in the expression of *MICA* (Figure S3A), *ULBP‐1* (Figure S3B), *ULBP‐2* (Figure S3C), and *ULBP‐3* (Figure S3D) in A549 cells when co‐cultured with NK‐92MI cells. Furthermore, aminoflavone 8 demonstrated the ability to suppress *PD‐L1* gene expression in A549 cells under co‐culture conditions (Figure S4). These findings prompted further investigation into the mechanisms underlying the observed effects.

STAT3 is a key transcription factor that is often constitutively activated in tumour cells, contributing to tumorigenesis, and in immune cells within the tumour microenvironment. Its activation is commonly mediated by cytokines, as well as growth factors, through the JAK/STAT signalling pathway [51]. In tumour cells, STAT3 promotes proliferation, immune evasion, and survival. Additionally, several studies have demonstrated that blocking STAT3 can enhance tumour surveillance by NK cells [39, 40, 41]. Researchers have previously reported that STAT3 activation plays a role in NK cell‐mediated tumour immune surveillance by regulating the expression of cytolytic effectors (such as granzymes or perforin) [52, 53], cytokines (such as IFN‐γ) [39, 52, 54, 55], natural cytotoxicity receptors and NKG2D [56], and ligands (such as MICA/B and ULBPs) [39] as well as immune checkpoint proteins (such as PD‐L1) [57, 58, 59]. The results in the current study indicate that the effects of aminoflavone 8 in inhibiting STAT3 phosphorylation could affect the expressions of cytotoxic effectors, surface markers, ligands, and immune checkpoints in NK‐92MI or A549 cells. Thus, we posit that aminoflavone 8 modulates the expressions of NK cytotoxic markers in NK or lung cancer cells by inhibiting STAT3 phosphorylation.

Previous studies reported that aminoflavone 8 targets cyclin‐dependent kinase 2 (CDK2), and the QUSA model indicated that the −OCH_3_ groups were favoured [60]. CDK2 is frequently overexpressed and hyperactivated in various cancers, making it a potential therapeutic target [61]. While the role of CDK2 in NK cells is less well characterised compared to its role in cancer cells, further investigation into its potential involvement in NK cell function could provide important insights into the mechanism of aminoflavone 8. Note that this was the first study to demonstrate that flavones 2, 3, and 6 and aminoflavone 8 can markedly enhance the cytotoxic activity of NK‐92MI cells against lung cancer cells without cytotoxic effects on MRC5 normal cells. It also appears that the effects of aminoflavone 8 involve modulating the expressions of cytokines, cytolytic effectors, receptors, and the corresponding ligands. In an animal model, aminoflavone 8 was shown to suppress tumour growth in lung cancer xenografts. Aminoflavone 8 was also shown to have a synergistic effect on the anti‐tumour effects of NK‐92MI cells in vivo without toxic effects on the kidney or liver. Taken together, it appears that this novel aminoflavone could be an immunostimulatory agent for NK cells, making it a promising candidate for tumour treatment.

## Author Contributions

**Ping‐Chih Hsu:** conceptualization (equal), data curation (equal), funding acquisition (equal), investigation (equal), resources (equal), writing – review and editing (equal). **Chih‐Cheng Chien:** data curation (equal), writing – review and editing (equal). **Yang‐Je Cheng:** methodology (equal), project administration (equal), resources (equal). **Chuan‐Hsin Chang:** conceptualization (equal), data curation (equal), formal analysis (equal), funding acquisition (equal), investigation (equal), methodology (equal), project administration (equal), resources (equal), visualization (equal), writing – original draft (equal), writing – review and editing (equal). **Tzenge‐Lien Shih:** conceptualization (equal), funding acquisition (equal), investigation (equal), methodology (equal), project administration (equal), resources (equal), supervision (equal), validation (equal), visualization (equal), writing – review and editing (equal).

## Ethics Statement

All protocols were approved by the Institutional Animal Care and Use Committee of Chang Gung Memorial Hospital (IACUC approval no. 202309201).

## Consent

All authors have given consent for publication.

## Conflicts of Interest

The authors declare no conflicts of interest.

## Supporting information


**Data S1:** jcmm71055‐sup‐0001‐DataS1.docx.

## Data Availability

The datasets used in the current study are available from the corresponding author upon reasonable request.

## References

[1] V. Uivarosi , A.‐C. Munteanu , and G. M. Nițulescu , “An Overview of Synthetic and Semisynthetic Flavonoid Derivatives and Analogues: Perspectives in Drug Discovery,” in Studies in Natural Products Chemistry, vol. 60 (Elsevier, 2019), 29–84, 10.1016/B978-0-444-64181-6.00002-4.

[2] A. Panche , A. Diwan , and S. Chandra , “Flavonoids: An Overview,” Nutrition Science 5, no. 5 (2016): e47, 10.1017/jns.2016.41. PMC5465813 28620474

[3] S. Liga , C. Paul , and F. Péter , “Flavonoids: Overview of Biosynthesis, Biological Activity, and Current Extraction Techniques,” Plants 12, no. 14 (2023): 2732, 10.3390/plants12142732. 37514347 PMC10384615

[4] E. Giannoni , F. Bianchini , L. Masieri , et al., “Reciprocal Activation of Prostate Cancer Cells and Cancer‐Associated Fibroblasts Stimulates Epithelial‐Mesenchymal Transition and Cancer Stemness,” Cancer Research 70, no. 17 (2010): 6945–6956, 10.1158/0008-5472.CAN-10-0785. 20699369

[5] M. A. Callero , G. V. Suárez , G. Luzzani , B. Itkin , B. Nguyen , and A. I. Loaiza‐Perez , “Aryl Hydrocarbon Receptor Activation by Aminoflavone: New Molecular Target for Renal Cancer Treatment,” International Journal of Oncology 41, no. 1 (2012): 125–134, 10.3892/ijo.2012.1427. 22485252

[6] M. J. Kuffel , J. C. Schroeder , L. J. Pobst , et al., “Activation of the Antitumor Agent Aminoflavone (NSC 686288) is Mediated by Induction of Tumor Cell Cytochrome P450 1A1/1A2,” Molecular Pharmacology 62, no. 1 (2002): 143–153, 10.1124/mol.62.1.143. 12065765

[7] N. M. Thorat , A. P. Sarkate , D. K. Lokwani , S. V. Tiwari , R. Azad , and S. R. Thopate , “N‐Benzylation of 6‐Aminoflavone by Reductive Amination and Efficient Access to Some Novel Anticancer Agents via Topoisomerase II Inhibition,” Molecular Diversity 25, no. 2 (2021): 937–948, 10.1007/s11030-020-10079-1. 32249379

[8] S. Ahmad , S. A. Shah , N. Khan , U. Nishan , N. Jamila , and A. Alotaibi , “A Phytoconstituent 6‐Aminoflavone Ameliorates Lipopolysaccharide‐Induced Oxidative Stress Mediated Synapse and Memory Dysfunction via p‐Akt/NF‐kB Pathway in Albino Mice,” Open Chemistry 21, no. 1 (2023): 20220336, 10.1515/chem-2022-0336.

[9] S. Ahmad , S. A. Shah , U. Nishan , et al., “6‐Aminoflavone Activates Nrf2 to Inhibit the Phospho‐JNK/TNF‐α Signaling Pathway to Reduce Amyloid Burden in an Aging Mouse Model,” ACS Omega 8, no. 30 (2023): 26955–26964, 10.1021/acsomega.3c01781. 37546603 PMC10399177

[10] M. A. Callero , C. E. Rodriguez , A. Sólimo , E. de Bal Kier Joffé , and A. I. Loaiza Perez , “The Immune System as a New Possible Cell Target for AFP 464 in a Spontaneous Mammary Cancer Mouse Model,” Journal of Cellular Biochemistry 118, no. 9 (2017): 2841–2849, 10.1002/jcb.25934. 28206673

[11] H. Sung , J. Ferlay , R. L. Siegel , et al., “Global Cancer Statistics 2020: GLOBOCAN Estimates of Incidence and Mortality Worldwide for 36 Cancers in 185 Countries,” CA: A Cancer Journal for Clinicians 71, no. 3 (2021): 209–249, 10.3322/caac.21660. 33538338

[12] L. Ye , J. Creaney , A. Redwood , and B. Robinson , “The Current Lung Cancer Neoantigen Landscape and Implications for Therapy,” Journal of Thoracic Oncology 16, no. 6 (2021): 922–932, 10.1016/j.jtho.2021.01.1624. 33581342

[13] X. Hu , H. Xu , Q. Xue , R. Wen , W. Jiao , and K. Tian , “The Role of ERBB4 Mutations in the Prognosis of Advanced Non‐Small Cell Lung Cancer Treated With Immune Checkpoint Inhibitors,” Molecular Medicine 27 (2021): 1–14, 10.1186/s10020-021-00387-z. 34620079 PMC8496027

[14] Z.‐F. Lim and P. C. Ma , “Emerging Insights of Tumor Heterogeneity and Drug Resistance Mechanisms in Lung Cancer Targeted Therapy,” Journal of Hematology & Oncology 12, no. 1 (2019): 134, 10.1186/s13045-019-0818-2. 31815659 PMC6902404

[15] X. Wang , H. Zhang , and X. Chen , “Drug Resistance and Combating Drug Resistance in Cancer,” Cancer Drug Resistance 2, no. 2 (2019): 141, 10.20517/cdr.2019.10. 34322663 PMC8315569

[16] W. M. Yokoyama , S. Kim , and A. R. French , “The Dynamic Life of Natural Killer Cells,” Annual Review of Immunology 22 (2004): 405–429, 10.1146/annurev.immunol.22.012703.104711. 15032583

[17] G. Trinchieri , “Biology of Natural Killer Cells,” Advances in Immunology 47 (1989): 187–376, 10.1016/s0065-2776(08)60664-1. 2683611 PMC7131425

[18] B. Smith , D. Rosenthal , and K. Ault , “Natural Killer Lymphocytes in Hairy Cell Leukemia: Presence of Phenotypically Identifiable Cells With Defective Functional Activity,” Experimental Hematology 13, no. 3 (1985): 189–193. 3884356

[19] F. Cui , D. Qu , R. Sun , M. Zhang , and K. Nan , “NK Cell‐Produced IFN‐γ Regulates Cell Growth and Apoptosis of Colorectal Cancer by Regulating IL‐15,” Experimental and Therapeutic Medicine 19, no. 2 (2020): 1400–1406, 10.3892/etm.2019.8343. 32010315 PMC6966233

[20] E. Gross , J. B. Sunwoo , and J. D. Bui , “Cancer Immunosurveillance and Immunoediting by Natural Killer Cells,” Cancer Journal 19, no. 6 (2013): 483–489, 10.1097/ppo.0000000000000005. 24270347

[21] D. H. Kaplan , V. Shankaran , A. S. Dighe , et al., “Demonstration of an Interferon γ‐Dependent Tumor Surveillance System in Immunocompetent Mice,” Proceedings of the National Academy of Sciences 95, no. 13 (1998): 7556–7561, 10.1073/pnas.95.13.7556. PMC22681 9636188

[22] J. Wu and L. L. Lanier , “Natural Killer Cells and Cancer,” Advances in Cancer Research 90, no. 1 (2003): 127–156, 10.1016/s0065-230x(03)90004-2. 14710949

[23] T. Sutlu and E. Alici , “Natural Killer Cell‐Based Immunotherapy in Cancer: Current Insights and Future Prospects,” Journal of Internal Medicine 266, no. 2 (2009): 154–181, 10.1111/j.1365-2796.2009.02121.x. 19614820

[24] L. Moretta and A. Moretta , “Unravelling Natural Killer Cell Function: Triggering and Inhibitory Human NK Receptors,” EMBO Journal 23, no. 2 (2004): 255–259, 10.1038/sj.emboj.7600019. 14685277 PMC1271745

[25] J. Russick , P.‐E. Joubert , M. Gillard‐Bocquet , et al., “Natural Killer Cells in the Human Lung Tumor Microenvironment Display Immune Inhibitory Functions,” Journal for Immunotherapy of Cancer 8, no. 2 (2020): e001054, 10.1136/jitc-2020-001054. 33067317 PMC7570244

[26] Y. Zhao , N. Chen , Y. Yu , et al., “Prognostic Value of MICA/B in Cancers: A Systematic Review and Meta‐Analysis,” Oncotarget 8, no. 56 (2017): 96384, 10.18632/oncotarget.21466. 29221214 PMC5707108

[27] S. Terry , A. Abdou , A. S. T. Engelsen , et al., “AXL Targeting Overcomes Human Lung Cancer Cell Resistance to NK‐ and CTL‐Mediated Cytotoxicity,” Cancer Immunology Research 7, no. 11 (2019): 1789–1802, 10.1158/2326-6066.Cir-18-0903. 31488404

[28] R. Okita , A. Maeda , K. Shimizu , Y. Nojima , S. Saisho , and M. Nakata , “Clinicopathological Relevance of Tumor Expression of NK Group 2 Member D Ligands in Resected Non‐Small Cell Lung Cancer,” Oncotarget 10, no. 63 (2019): 6805–6815, 10.18632/oncotarget.27308. 31827723 PMC6887580

[29] T.‐L. Shih , C.‐E. Chou , W.‐Y. Liao , and C.‐A. Hsiao , “Copper‐Mediated Trimethylsilyl Azide in Amination of Bromoflavonoids to Synthesize Unique Aminoflavonoids,” Tetrahedron 70, no. 23 (2014): 3657–3664, 10.1016/j.tet.2014.04.022.

[30] P. Prayong , S. Barusrux , and N. Weerapreeyakul , “Cytotoxic Activity Screening of Some Indigenous Thai Plants,” Fitoterapia 79, no. 7–8 (2008): 598–601, 10.1016/j.fitote.2008.06.007. 18664377

[31] S. Thongnest , P. Chawengrum , S. Keeratichamroen , et al., “Vernodalidimer L, a Sesquiterpene Lactone Dimer From Vernonia Extensa and Anti‐Tumor Effects of Vernodalin, Vernolepin, and Vernolide on HepG2 Liver Cancer Cells,” Bioorganic Chemistry 92 (2019): 103197, 10.1016/j.bioorg.2019.103197. 31445193

[32] Y. X. Chen , C. H. Chang , C. W. Li , J. J. Chen , and T. L. Shih , “Design, Synthesis, and Evaluation of 1, 2, 3‐Triazole‐Based Benzenesulfonamide and Flavonol Hybrid Molecules as Anticancer Agents,” Journal of the Chinese Chemical Society 70, no. 10 (2023): 1924–1936, 10.1002/jccs.202300279.

[33] National Research Council Committee for the Update of the Guide for the C, Use of Laboratory A , “The National Academies Collection: Reports Funded by National Institutes of Health,” in Guide for the Care and Use of Laboratory Animals (National Academies Press, 2011).

[34] G. Z. Tau , S. N. Cowan , J. Weisburg , N. S. Braunstein , and P. B. Rothman , “Regulation of IFN‐γ Signaling Is Essential for the Cytotoxic Activity of CD8+ T Cells,” Journal of Immunology 167, no. 10 (2001): 5574–5582. 10.4049/jimmunol.167.10.5574 PMC4416493 11698428

[35] N. R. Maimela , S. Liu , and Y. Zhang , “Fates of CD8+ T Cells in Tumor Microenvironment,” Computational and Structural Biotechnology Journal 17 (2019): 1–13, 10.1016/j.csbj.2018.11.004. 30581539 PMC6297055

[36] C. Berthou , J. F. Bourge , Y. Zhang , et al., “Interferon‐Gamma‐Induced Membrane PAF‐Receptor Expression Confers Tumor Cell Susceptibility to NK Perforin‐Dependent Lysis,” Blood 95, no. 7 (2000): 2329–2336. 10733503

[37] S. E. Street , E. Cretney , and M. J. Smyth , “Perforin and Interferon‐γ Activities Independently Control Tumor Initiation, Growth, and Metastasis,” Blood 97, no. 1 (2001): 192–197, 10.1182/blood.V97.1.192. 11133760

[38] M. F. van den Broek , D. Kägi , R. M. Zinkernagel , and H. Hengartner , “Perforin Dependence of Natural Killer Cell‐Mediated Tumor Control In Vivo,” European Journal of Immunology 25, no. 12 (1995): 3514–3516, 10.1002/eji.1830251246. 8566046

[39] X. Sun , Q. Sui , C. Zhang , Z. Tian , and J. Zhang , “Targeting Blockage of STAT3 in Hepatocellular Carcinoma Cells Augments NK Cell Functions via Reverse Hepatocellular Carcinoma–Induced Immune Suppression,” Molecular Cancer Therapeutics 12, no. 12 (2013): 2885–2896, 10.1158/1535-7163.MCT-12-1087. 24107450

[40] Q. Sui , J. Zhang , X. Sun , C. Zhang , Q. Han , and Z. Tian , “NK Cells Are the Crucial Antitumor Mediators When STAT3‐Mediated Immunosuppression Is Blocked in Hepatocellular Carcinoma,” Journal of Immunology 193, no. 4 (2014): 2016–2023, 10.4049/jimmunol.1302389. 25015826

[41] T.‐L. Hwang and C.‐H. Chang , “Oridonin Enhances Cytotoxic Activity of Natural Killer Cells Against Lung Cancer,” International Immunopharmacology 122 (2023): 110669, 10.1016/j.intimp.2023.110669. 37480753

[42] J. H. Gong , G. Maki , and H. G. Klingemann , “Characterization of a Human Cell Line (NK‐92) With Phenotypical and Functional Characteristics of Activated Natural Killer Cells,” Leukemia 8, no. 4 (1994): 652–658. 8152260

[43] H. Klingemann , L. Boissel , and F. Toneguzzo , “Natural Killer Cells for Immunotherapy–Advantages of the NK‐92 Cell Line Over Blood NK Cells,” Frontiers in Immunology 7 (2016): 170692, 10.3389/fimmu.2016.00091. PMC4789404 27014270

[44] T. Tonn , D. Schwabe , H. G. Klingemann , et al., “Treatment of Patients With Advanced Cancer With the Natural Killer Cell Line NK‐92,” Cytotherapy 15, no. 12 (2013): 1563–1570, 10.1016/j.jcyt.2013.06.017. 24094496

[45] T. Tonn , S. Becker , R. Esser , D. Schwabe , and E. Seifried , “Cellular Immunotherapy of Malignancies Using the Clonal Natural Killer Cell Line NK‐92,” Journal of Hematotherapy & Stem Cell Research 10, no. 4 (2001): 535–544, 10.1089/15258160152509145. 11522236

[46] M. Cheng , Y. Chen , W. Xiao , R. Sun , and Z. Tian , “NK Cell‐Based Immunotherapy for Malignant Diseases,” Cellular & Molecular Immunology 10, no. 3 (2013): 230–252, 10.1038/cmi.2013.10. 23604045 PMC4076738

[47] G. Maki , H. G. Klingemann , J. A. Martinson , and Y. K. Tam , “Factors Regulating the Cytotoxic Activity of the Human Natural Killer Cell Line, NK‐92,” Journal of Hematotherapy & Stem Cell Research 10, no. 3 (2001): 369–383, 10.1089/152581601750288975. 11454312

[48] Y. K. Tam , G. Maki , B. Miyagawa , B. Hennemann , T. Tonn , and H. G. Klingemann , “Characterization of Genetically Altered, Interleukin 2‐Independent Natural Killer Cell Lines Suitable for Adoptive Cellular Immunotherapy,” Human Gene Therapy 10, no. 8 (1999): 1359–1373, 10.1089/10430349950018030. 10365666

[49] R. W. McGilvray , R. A. Eagle , P. Rolland , I. Jafferji , J. Trowsdale , and L. G. Durrant , “ULBP2 and RAET1E NKG2D Ligands Are Independent Predictors of Poor Prognosis in Ovarian Cancer Patients,” International Journal of Cancer 127, no. 6 (2010): 1412–1420, 10.1002/ijc.25156. 20054857

[50] Y. Zhang , C. Han , E. Shao , L. Sun , and D. Liu , “Expression, Prognosis, and Regulation of ULBP1, ULBP2, and ULBP3 in Human Breast Cancer,” 2021, 10.21203/rs.3.rs-574086/v1.

[51] H. Yu , D. Pardoll , and R. Jove , “STATs in Cancer Inflammation and Immunity: A Leading Role for STAT3,” Nature Reviews Cancer 9, no. 11 (2009): 798–809, 10.1038/nrc2734. 19851315 PMC4856025

[52] D. Gotthardt , E. M. Putz , E. Straka , et al., “Loss of STAT3 in Murine NK Cells Enhances NK Cell‐Dependent Tumor Surveillance,” Blood 124, no. 15 (2014): 2370–2379, 10.1182/blood-2014-03-564450. 25185262

[53] D. Gotthardt and V. Sexl , “STATs in NK‐Cells: The Good, the Bad, and the Ugly,” Frontiers in Immunology 7 (2016): 694, 10.3389/fimmu.2016.00694. 28149296 PMC5241313

[54] R. Bedel , A. Thiery‐Vuillemin , C. Grandclement , et al., “Novel Role for STAT3 in Transcriptional Regulation of NK Immune Cell Targeting Receptor MICA on Cancer Cells,” Cancer Research 71, no. 5 (2011): 1615–1626, 10.1158/0008-5472.Can-09-4540. 21257710

[55] N. A. Cacalano , “Regulation of Natural Killer Cell Function by STAT3,” Frontiers in Immunology 7 (2016): 128, 10.3389/fimmu.2016.00128. 27148255 PMC4827001

[56] S. Zhu , P. V. Phatarpekar , C. J. Denman , et al., “Transcription of the Activating Receptor NKG2D in Natural Killer Cells Is Regulated by STAT3 Tyrosine Phosphorylation,” Blood 124, no. 3 (2014): 403–411, 10.1182/blood-2013-05-499707. 24891320 PMC4102712

[57] T. L. Song , M. L. Nairismägi , Y. Laurensia , et al., “Oncogenic Activation of the STAT3 Pathway Drives PD‐L1 Expression in Natural Killer/T‐Cell Lymphoma,” Blood 132, no. 11 (2018): 1146–1158, 10.1182/blood-2018-01-829424. 30054295 PMC6148343

[58] V. Atsaves , N. Tsesmetzis , D. Chioureas , et al., “PD‐L1 Is Commonly Expressed and Transcriptionally Regulated by STAT3 and MYC in ALK‐Negative Anaplastic Large‐Cell Lymphoma,” Leukemia 31, no. 7 (2017): 1633–1637, 10.1038/leu.2017.103. 28344319

[59] M. Marzec , Q. Zhang , A. Goradia , et al., “Oncogenic Kinase NPM/ALK Induces Through STAT3 Expression of Immunosuppressive Protein CD274 (PD‐L1, B7‐H1),” Proceedings of the National Academy of Sciences of the United States of America 105, no. 52 (2008): 20852–20857, 10.1073/pnas.0810958105. 19088198 PMC2634900

[60] L. Simon , A. Imane , K. Srinivasan , L. Pathak , and I. Daoud , “In Silico Drug‐Designing Studies on Flavanoids as Anticolon Cancer Agents: Pharmacophore Mapping, Molecular Docking, and Monte Carlo Method‐Based QSAR Modeling,” Interdisciplinary Sciences: Computational Life Sciences 9 (2017): 445–458, 10.1007/s12539-016-0169-4. 27059855

[61] T. Chohan , H. Qian , Y. Pan , and J.‐Z. Chen , “Cyclin‐Dependent Kinase‐2 as a Target for Cancer Therapy: Progress in the Development of CDK2 Inhibitors as Anti‐Cancer Agents,” Current Medicinal Chemistry 22, no. 2 (2014): 237–263, 10.2174/0929867321666141106113633. 25386824

