# Supplementary material for: Flavones and Aminoflavones Increase the Cytotoxicity of NK Cells in Human Non‐Small Cell Lung Cancer
Source: J Cell Mol Med. 2026 Feb 13;30(4):e71055. doi: 10.1111/jcmm.71055 (PMC12904836; doi:10.1111/jcmm.71055)
Supplement: Supplementary file 1 — Data S1: jcmm71055‐sup‐0001‐DataS1.docx. [file JCMM-30-e71055-s001.docx]

Table S1. Forward and reverse primer sequences of transcripts used in Real-Time PCR.

| **Transcript** | Forward primer | Reverse primer |
| --- | --- | --- |
| GAPDH | ACCCACTCCTCCACCTTTGAC | TCCACCACCCTGTTGCTGTAG |
| Perforin | GGTTCACTGCCACGGATG | ACAGGTGCCAAGGAGGTC |
| Granzyme B | CTGATACGAGACGACTTC | GGATTATAGGCTGGATGG |
| IFN-γ | AGCTCTGCATCGTTTTGGGTT | GTTCCATTATCCGCTACATCTGAA |
| NKG2D | GGCTCATTCTCTCACCCA | TAAAGCTCGAGGCATAGAGT GC |
| MICA | ACTGCTTGAGCCGCT GAGA | GAGGTGCAAAAGGGAAGATGC |
| ULBP1 | CAGCAGACGATGAGGACATT | GACAGAAAGTGGCAGAAGGTG |
| ULBP2 | CATTACTTCTCAATGGGAGA CTGT | TGTGCCTGAGGACATGGCGA |
| ULBP3 | ATTCTTCCGTACCTGCTATT | GCTATCCTTCTCCCACTTCT |
| PD-L1 | GCTATGGTGGTGCCGACTAC | TTGGTGGTGGTGGTCTTACC |

**Figure S1**. Aminoflavone prodrugs for clinical trial.


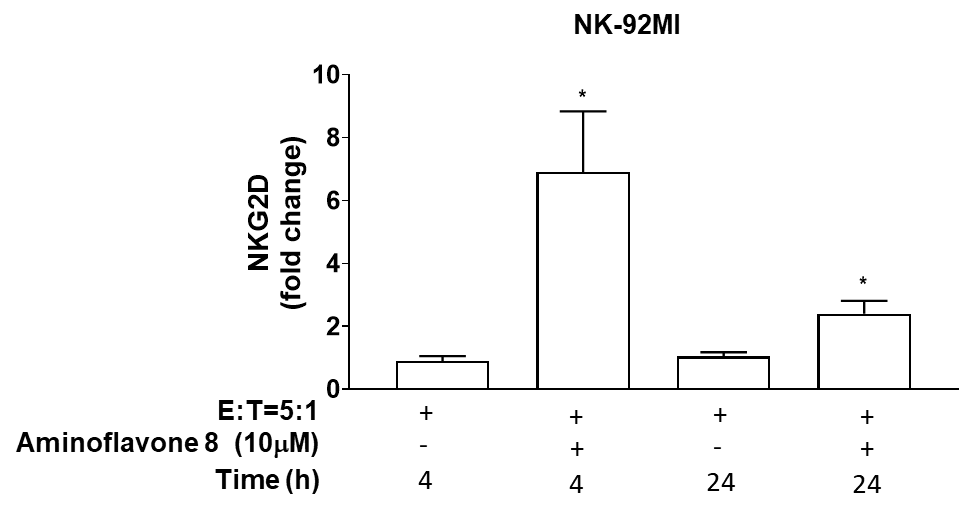


**Figure S2. The gene expression of *NKG2D* receptor in NK cells.** NK-92MI cells were co-cultured with aminoflavone **8**-pretreated A549 lung cancer cell lines at 10 μM (E:T ratio= 5:1) for either 4 or 24 h. The mRNA level of *NKG2D* was determined by qRT-PCR and normalized to the amount of *GAPDH* mRNA. The bars in the figure represent the mean ± SE, and statistical significance (**p* < 0.05) was assessed against the Model group.


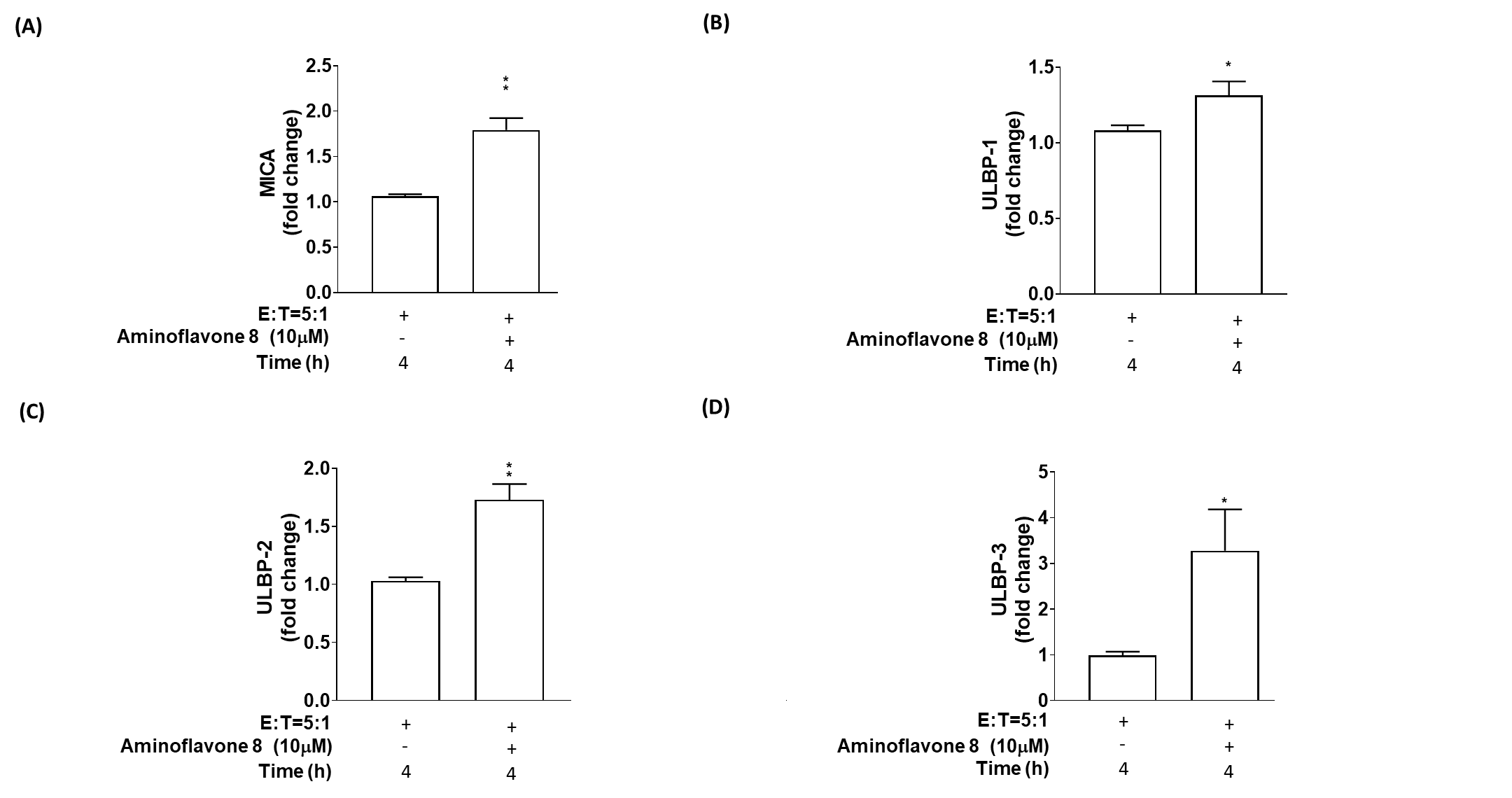


**Figure S3. The increased expressions of the ligands of NKG2D receptor in A549 cells upon the treatment of aminoflavone 8.** A549 cells were pretreated with aminoflavone **8** (10 μM) and co-culture with NK-92MI cells at E: T ratio=5:1. mRNA expression of *MICA* (A), *ULBP-1* (B), *ULBP-2* (C), and *ULBP-3* (D) were determined by qRT-PCR and normalized to the amount of *GAPDH* mRNA. The bars in the figure represent the mean ± SE, and statistical significance (**p* < 0.05; ***p* < 0.01) was assessed against the Model group.


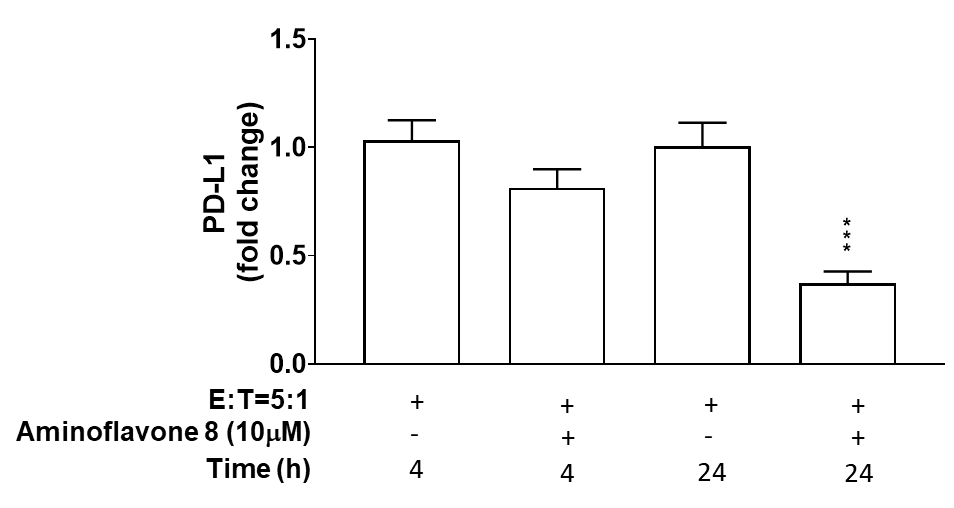


**Figure S4. The gene expressions of *PD-L1* in A549 lung cancer cells.** A549 lung cancer cells were treated with aminoflavone **8** or co-cultured with NK-92MI cells at an effector-to-target ratio of 5:1 (E/T=5:1). Furthermore, A549 lung cancer cells were pre-treated with aminoflavone **8** and co-cultured with NK-92MI cells at the same E/T ratio (5:1) for either 4 or 24 h. The mRNA levels of *PD-L1* were determined by qRT-PCR and normalized to the amount of *GAPDH* mRNA. The bars in the figure represent the mean ± SE, and statistical significance (****p* < 0.0001) was assessed against the Model group.
